# Tumor morphological evolution: directed migration and gain and loss of the self-metastatic phenotype

**DOI:** 10.1186/1745-6150-5-23

**Published:** 2010-04-20

**Authors:** Heiko Enderling, Lynn Hlatky, Philip Hahnfeldt

**Affiliations:** 1 Center of Cancer Systems Biology, St Elizabeth's Medical Center, Tufts University School of Medicine, Boston, MA 02135, USA

## Abstract

**Background:**

Aside from the stepwise genetic alterations known to underlie cancer cell creation, the microenvironment is known to profoundly influence subsequent tumor development, morphology and metastasis. Invasive cluster formation has been assumed to be dependent on directed migration and a heterogeneous environment - a conclusion derived from complex models of tumor-environment interaction. At the same time, these models have not included the prospect, now supported by a preponderance of evidence, that only a minority of cancer cells may have stem cell capacity. This proves to weigh heavily on the microenvironmental requirements for the display of characteristic tumor growth phenotypes. We show using agent-based modeling that some defining features of tumor growth ascribed to directed migration might also be realized under random migration, and discuss broader implications for cause-and-effect determination in general.

**Results:**

Considering only the properties of random migration in tumors composed of stem cells and committed cells, we are able to recapitulate a characteristic clustering feature of invasive tumor growth, a property we attribute to "self-metastatic" growth. When the additional influence of directed migrations under chemotactic environments are considered, we find that tumor growth and invasive morphology are supported while the tumor is distant from the source, but are progressively discouraged as the tumor converges about that source.

**Conclusions:**

We show that invasive clustering can derive from basic kinetic assumptions often neglected in more complex models. While higher-order mechanisms, e.g. directed migration upon chemotactic stimuli, may result in clustering growth morphologies, exclusive attributions of this phenotype to this or other structured microenvironments would be inappropriate, in light of our finding these features are observable in a homogeneous environment. Furthermore, directed migration will result in loss of the invasive phenotype as the tumor approaches the attractor source. Reviewers: This article was reviewed by Mark Little and Glen Webb.

## Background

Cancer development and tumor growth are complex dynamic phenomena whose spatio-temporal evolution depends on intrinsic properties of the cancer cells as well as on environmental factors. Among the environmental dependencies, the angiogenic switch is probably the most well established bottleneck a tumor has to overcome before a tumor can progress to clinical disease [1,2].

**Figure 1:**
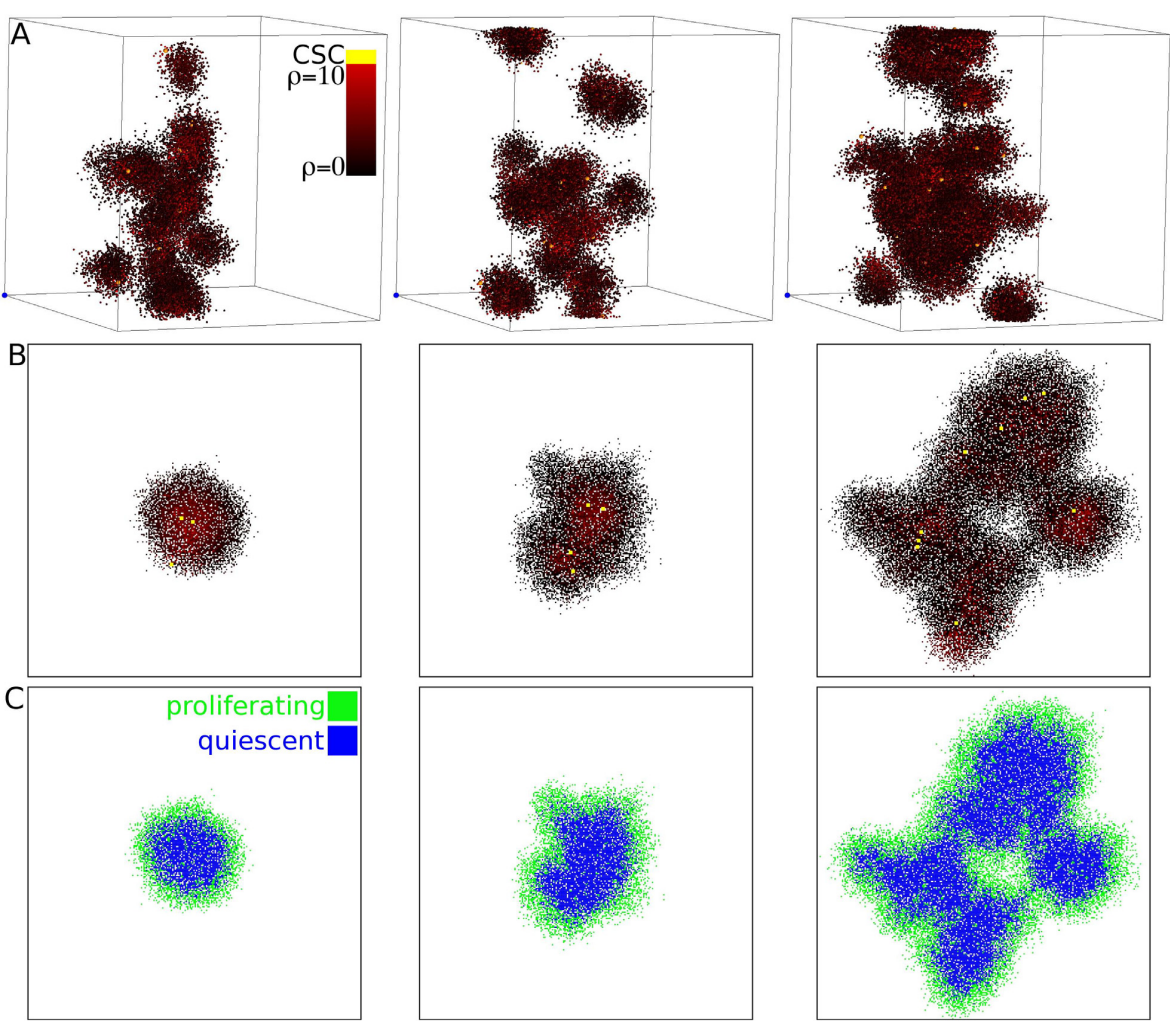
**Random motility and self-metastatic growth**. A) Visualization of self-metastatic growth at three different time points in three-dimensional space. Individual tumor clusters are driven by cancer stem cells (yellow), and each cluster features a radial proliferation capacity fall off (red to black). B) Self-metastatic growth in two-dimensional space. C) Distribution of proliferating and quiescent cells in the tumor populations shown in B. *Other parameters are μ *= *15, p*_*s *_= *1*.

For many years, cancer cells have been defined by six hallmarks - self-sufficiency in growth signals, insensitivity to anti-growth signals, evading apoptosis, limitless replicative potential, sustained angiogenesis and tissue invasion and metastasis [3]. Challenging this uniform model of cancer is the emerging cancer stem cell hypothesis, which assumes contrarily that only a minority of cells - the so-called cancer stem cells - possesses all these hallmarks [4,5]. The remainder, being mortal and having a limited proliferation capacity, do not present a sustainable threat to the host, but nevertheless complicate tumor growth dynamics in counterintuitive ways not anticipated by all cells possessing the classic hallmarks. One might argue that it is the abundant presence of non-stem cells that gives the stem cells their unique features - when all cells are stem cells, as is commonly assumed in current models, tumors grow almost homogeneously in a radially symmetric manner [6-8]. We have previously shown that the spatio-temporal evolution of tumors driven by a minority cancer stem cell population follows a distinct pattern - self-metastatic growth [9]. This invasive phenotype develops from the interplay of intrinsic tumor mechanisms - tumor cell proliferation, random motility and cell death. As cell crowding inhibits cancer cell proliferation [10], tumor expansion without corresponding liberation into free space will slow tumor growth overall. By contrast, liberation of stem cells, achieved by random cell motility and death among their non-stem progeny, allows the continual seeding of clones in the tumor periphery that contribute to the efficient expansion of the tumor. The emerging tumor population is described as conglomerates of self-metastatic clones - each clones driven by stem cells and proliferation confined mostly to the outer rim (Figure 1).

Here we show that the overall effect of the microenvironment on tumor growth may be largely anticipated by its influence on migration-enabled seeding of new clones at the tumor periphery. In particular we show a biphasic impact of directed migration up gradients in the extracellular matrix. Directed migration yields liberation of cancer stem cells in tumor clusters as progeny continuously migrate away and thus release space constraints. However, as a tumor approaches the source of the gradient, clustering away from the tumor is discouraged and tumor growth slows.

## Results

**Figure 2:**
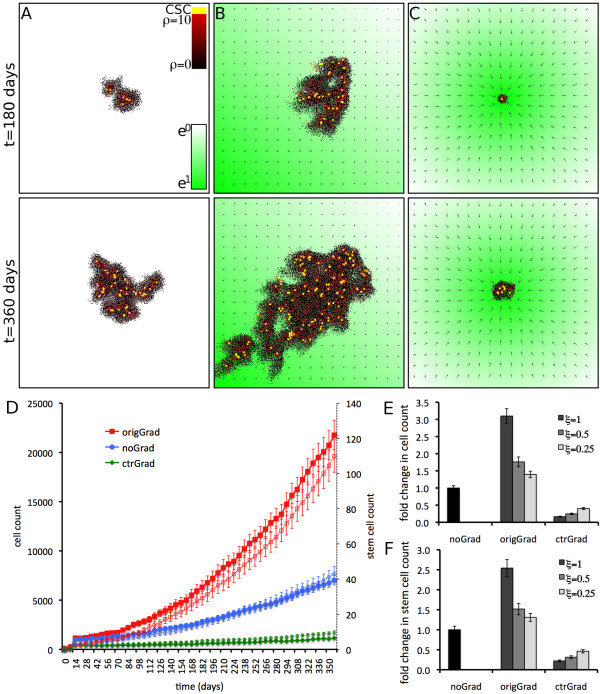
**Taxis dependent tumor size and morphology**. Tumor growth simulated for t = 360 days from a single cancer stem cell located the center of the computational domain (A, C) or in the center of the top right quarter P(width*3/4, height*3/4) (B). The strength of the attractor is shown in green, and green arrows show the direction and strength of local gradients. Yellow cells are cancer stem cells, and red-black shows the cellular remaining proliferation capacity *ρ*. A) Homogeneous domain, random motility only. B) Attractor at the origin of the computational domain P(0,0), *ξ *= 1. C) Attractor at center of the computational domain P(width/2, height/2), *ξ *= 1. D) Change in cell number over time for n = 25 simulations each of tumor growth in domains without gradients (blue circles), center gradient (green diamonds) and origin gradient (red squares). Respective numbers of stem cells are shown as thin plots (open markers). Shown are averages and standard error. E) Fold change in cell count after t = 360 days in different domains for various chemotactic response strengths *ξ*. F) Fold change in stem cell count after t = 360 days in different domains for various chemotactic response strengths *ξ*. *Other parameters are ρ *= *10, μ *= *5, p*_*s *_= *10*.

Cellular migration in response to environmental gradients can be subdivided into two different phases, depending on the position of cells in the gradient field. We initiate simulations of tumor growth with single cells being located near and far from the source to compare the impact of directed migration on tumor morphology to dynamics solely dependent on random motility (Figures 1, 2A).

### Directed migration towards an attractor source

We position an attractor source at the origin of the computational domain of width *W *and height *H *(bottom left corner, (*0,0*)) with an exponentially decreasing gradient as either x or y is increased.

and the initial cancer stem cell being located in the center of the top-right quadrant of the domain at P(W*3/4, H*3/4). We simulate tumor growth for t = 360 days in 25 independent simulations for different taxis response probabilities *G *= 1 - exp(-*ξz*)), which apply above and beyond random migration (see Methods for details). Here, *z *is the product of || and another factor related to the available sites *S *and the angular direction of the gradient relative to those sites. The parameter *ξ *is the strength of the relationship between the value of *z *and the probability of directed cell movement - the chemotactic responsiveness of a cell.

Comparable to simulations of random motility alone, initially a small tumor cluster forms around the seeded cancer stem cell. When directed migration is strong (*ξ *= 1), the offspring continuously migrate towards the attractor, which has the effect of further loosing space constraints in the primary tumor cluster (Figure 2B). As a result, the cancer stem cells proliferate frequently. Here and throughout, it is assumed that when stem cells divide, they reproduce themselves by "symmetric division" at a (low) frequency p_s _(estimated to be = 1% of stem cell divisions). The rest of the time, their divisions are assumed to be asymmetric, producing a stem and a non-stem cell. Non-stem cells divide until their generational lifetimes are exhausted, here taken to be *ρ *= 10 divisions. For *ξ *= 1, the tumors after t = 360 days consist on average of 21,765 ± 1,505 cells (110 ± 9 cancer stem cells), a significant increase (*p *< 10^-6^) compared to tumors developing with random motility alone (7,023 ± 484 (43 ± 4) cells, Figure 2D). If the contribution of directed migration to tumor growth and morphology evolution is lowered to *ξ *= 0.5, yields approaching the tumor growth rates and morphologies observed without directed migration are obtained (12,374 (66) and 9,808 (56) cells, respectively, Figure 2E).

### Directed migration at an attractor source

We position an attractor source at the center of the computational domain (*W*/2, *H*/2) with a radially exponentially decreasing gradient , where:

and the initial cancer stem cell is located right at the attractor source. We simulate tumor growth for t = 360 days in 25 independent simulations for different taxis response strengths *ξ*.

Comparable to simulations of random motility alone, initially a small tumor cluster forms around the seeded cancer stem cell. A strong directed gradient with *ξ *= 1 towards the attractor source and the tumor core results in a continuous inbound migration of cells on the periphery. This results in a persistent spatial inhibition of cancer stem cells in the core of the cluster (Figure 2C). The cancer stem cells divide only infrequently, and the tumor population does not grow. After t = 360 days the tumors consist on average of 1,151 ± 102 cells (10 ± 1 cancer stem cells), a significant decrease (*p *< 10^-13^) compared to tumors developing without directed migration solely dependent on random motility (Figure 2D). If the contribution of directed migration to tumor morphology evolution is lowered to *ξ *= 0.5 and *ξ *= 0.25, yields approaching the tumor growth rates and morphologies observed without directed migration (1,723 (14) and 2,822 (20)) cells, respectively (Figure 2F) are again observed.

### Biphasic influence of directed migration

**Figure 3:**
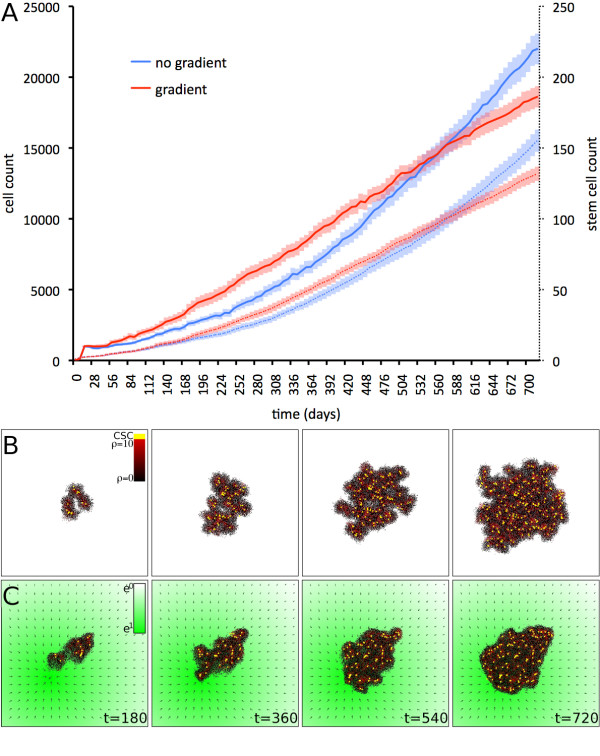
**Biphasic influence of directed migration**. Comparison of simulations of tumor growth without environmental gradients (random migration only; blue plots) to tumor growth with directed migration up a gradient to a nearby attractor (red plots). A) During directed migration towards an attractor source the tumor grows faster due to continuous stem cell liberation. Once the tumor has reached the attractor source intra-tumoral competition for space due to inbound directed migration slows down tumor growth. The biphasic influence is observed in total cell count (solid lines) and stem cell count (dotted lines). B) Representative simulation of self-metastatic tumor growth without environmental gradients. C) Representative simulation of tumor growth initiated at P(width*1/3, height*1/3) with directed migration towards an attractor source at P(width*2/3, height*2/3). The strength of the attractor is shown in green, and green arrows show the direction and strength of local gradients. An initial growth phase towards the attractor is followed by growth around the attractor. *Other parameters are ρ *= *10, μ *= *5, p*_*s *_= *10*.

We now combine migration towards an attractor and migration of cells at an attractor source in single simulations to understand cellular migration in response to environmental gradients. Initially, cells distant from the attractor source move in a directed manner towards the attractor resulting in a widespread morphology of the population. In the second phase the cells are located around the attractor source, and the continuing migration of the cells towards the attractor, and thus into the cluster, yields a compact morphology. With competition for space and cellular migration being the dominant determinants of tumor progression and morphological evolution [9,11], we report a biphasic behavior for the overall effect of directed migration on tumor growth and morphology. Initial directed migration of cells distant to an attractor promotes tumor growth due to liberation of intra-tumoral space, but as the growing tumor cluster reaches the attractor source, tumor growth is slowed down due to inbound migration and continuous space inhibition (Figure 3). The growth curves of these tumors and tumours growing without gradients therefore intersect, a feature also observed in the stem cell counts.

## Discussion

Tumors that consist of stem cells and non-stem progenitors and develop with random motility alone, i.e. without environmental gradients, exhibit a self-metastatic phenotype. Stem cells are spatially inhibited by their mortal progeny and thus can only form clusters of a limited size. For tumor progression, space has to be freed around the stem cell to allow for symmetric division and migration to seed a new cluster nearby.

Here we presented a study of the impact of cell migration directed by environmental gradients on a growing tumor population. The continuous migration of tumor cells away from the tumor boundary and towards an attractor source relaxes spatial constraints within tumor clusters, which in turn facilitates increased stem cell division and self-metastatic seeding. If the tumor cluster is sufficiently far away from the attractor, directed migration significantly promotes tumor growth. On the other hand, if the tumor cluster is located around the attractor source, preferred inbound migration introduces spatial constraints on the tumor cluster and the stem cells within. This will result in loss of stem cell division, loss of the self-metastatic phenotype and significantly reduced tumor growth. Depending on the position of a developing tumor cluster to an attractor, directed migration can therefore have a biphasic impact on tumor growth.

We would not expect second-order effects relating to gradients to change these findings. Random perturbations to either gradient strength or cell responsiveness, such as may occur from enzymatic degradations or cell mutations, respectively, would be expected to increase random cell drift away from the tumor mass (if the source is at a distance from the tumor), as there are fewer random ways for cells to compact together than there are for them to drift apart. For this reason it would be expected that undirected second-order effects modulating chemotactic gradients would produce more migration overall, and thus, as here shown, to accelerated tumor growth.

## Conclusions

Directed migration of a heterogeneous population of tumor stem cells and progenitor cells in response to environmental gradients alters tumor growth rate as well as intrinsic tumor morphology obtained by random cell motility alone. Dependent on the relative position of tumors to the attractor source tumor growth is promoted or inhibited, and the intrinsic self-metastatic morphology can be enhanced or completely lost. When analyzing the dynamic behavior of a tumor population, attention should be paid to what morphologies and growth rates are due to the intrinsic properties of tumor cells, and which reflect the environment where the tumor develops. By using minimally-parameterized models that better capture fundamental tumor compositions and kinetics, one may, as here, uncover properties intrinsic to tumor cells that have already been attributed by other models to more complex environmental factors. With the improved understanding of the role of intrinsic factors gained from these models, we might more reliably account for how environmental perturbations really alter the overall tumor growth dynamic.

## Methods

We use an agent-based computer model [12,13] to simulate single cell dynamics, and derive complex population dynamics from cells interacting with each other and their environment. Model details have been previously published [9,14]. Briefly, in line with the cancer stem cell hypothesis we assume all cancer cells being able to proliferate a certain number of times, *ρ*_max_, before dying and thus ultimately being removed from the simulation. For cancer stem cells, we assume *ρ*_max _= ∞. Cells need to mature through the cell cycle before division can occur, which takes cell type dependent time, in our simulations set to be *τ *= 24 hours. Dependent on available space cells can migrate with rate *μ*. Without available space, i.e. none of the adjacent eight lattice points on a two-dimensional grid is vacant, we assume the cell to be inhibited by their neighboring cells and forced to rest in a quiescent state until again exposed to space. We initiate a single cell in the center of our 3,500 *μ*m × 3,500 *μ*m computational domain, composed of 350^2 ^equal-sized blocks of 100 *μ*m^2 ^each that can hold at the most one cell at any time. By simulating cell proliferation, migration and cell death at discrete time intervals Δ*t*, complex population behaviors emerge. The dynamics of these populations can be observed by tracking the number of quiescent and proliferating cells over time, as well as their spatio-temporal morphological evolution.

### Random motility

We will consider two cellular migration strategies - random motility and directed migration up gradients in the extra-cellular matrix. Without environmental attractors the cells perform a random walk, and the decision of a cell at current position (*x*_0_, *y*_0_) where to move to is based upon available lattice points in the immediate neighborhood *N*_(*x*0, *y*0)_. The probabilities are calculated according to the following rule:

With probability 1/(1+number of unoccupied neighboring lattice points) the cell remains temporarily stationary. If a cell is completely surrounded by other cells the probability of not moving is the only choice.

### Directed migration

Let *F *be a function whose gradient  describes a chemotactic stimulus due to the distribution of an attractant in the extracellular matrix. Let  be the vector connecting the center of the lattice point (*x*_0_, *y*_0_) where a cell resides at time *t*, to the center of one of the eight adjacent lattice points (*x*_*i*_, *y*_*i*_). We now consider the values of  in each surrounding lattice point that are *positive *and correspond to an unoccupied site. We call these the Available Sites (*S*). Note that migrations in negative directions do not occur as *directed *movements. Of the *S *nontrivial movements, we weight each by the factor cos(angle *θ *between the gradient  and the movement direction  for each possible movement), for the sake of calculating the angle-weighted relative opportunities for movement in each available direction. The cos(*θ*) are summed over the *S *available sites to get *AS *(*A *is the average cosine value). Treating the effective "cos(*θ*)" of non-movement to be 1, the total sum of cosines is *AS*+1, where *AS *corresponds to actual movement. The total angle-weighted relative opportunity for movement (to one of the *S *sites) due to directed migration is thus *AS*/(*AS*+1) where *A *is the average of the cos(*θ*) over these sites. This value, times the gradient strength ||, is now mapped to a probability of mobilization *G *through an arbitrary chemotactic/haptotactic responsiveness function *H*(*z*) for the cells, defined such that *H*(0) = 0 and *H *→ 1 monotonically as *z *→ ∞. The result is *G *= *H*{ ||*AS*/(*AS*+1)}. *G *is the probability the gradient succeeds in mobilizing the cell, and takes into account the proportion of movement possible and the strength of the gradient. Once mobilized, where the cell then goes is weighted by the respective cosines for movement, so that cos(*θ*)*G*/(*AS*) is the probability of movement to the available site at angle *θ*. We chose *H*(*z*) = 1 - exp(-*ξz*), where *ξ *is a parameter that scales the contribution of [(gradient strength) × (movement opportunity)] to the actual probability of cell movement.

Algorithmically, directed migrations are considered at the same frequency *μ *as random migrations, where *μ *= 0.00635 mm h^-1 ^or about 15 lattice moves (cell widths) per day [15]. For weak local gradients a cell is less likely to move at all due to the gradient than for strong local gradients, so random movements will be the only reason for movement and will thus dominate. By contrast, for strong gradients, the probability of directed movement approaches 1 when favorable lattice points are available, so it will compete strongly with the random process.

## Competing interests

The authors declare that they have no competing interests.

## Authors' contributions

PH and HE developed the idea for this study. PH derived the equations for directed migration. HE implemented and performed the simulations. PH, LH, HE wrote the manuscript.

## Reviewers' Comments

### Reviewer 1: Mark Little, Imperial College Faculty of Medicine, London, UK

#### General comments

This is an interesting and generally clearly-written paper, although short of methodological detail. Arguably, the model presented is merely schematic, useful nevertheless as illustrating the idea that random+directed motility could aid tumour growth, at least in early stages of tumour development. The detailed mathematical assumptions made (e.g., in relation to the expressions on pp.10-11 describing the chemo-taxis process in the Methods) I suspect have no biological basis, even more so the particular parameter values assumed. What is missing is any biological justification of the gradient-following mechanism. Do tumour cells follow-gradients?

There are a few missing, or poorly explained, things in the model, such as the relation between cancer stem cells and other tumour cells. [Perhaps I missed this somewhere.] I assume that most of the progeny of cancer stem cells are non-stem cells, but clearly there must be some proliferation of stem cells for the model to give the results it does.

#### Specific comments (page/line)

p.4 l.-6 Should this be "source of the gradient, clustering"?

p.10-11 I guess this chemotaxis process is equivalent to the standard Keller-Segel model [16,17] but this should be referenced.

p.11 l.6 What is the point of the *S*/(*S*+1) term in this expression? It is similar to that in the expression at end of 2nd para on p.10? However, the point in the expression on p.10 was clear (to make the probabilities sum to 1). These *G *do not sum to 1, so I assume they are renormalized to make sure that this is the case. Also, where has the  vector gone?

### Authors' Response

The conclusions are surprisingly robust over substantial variations in parameters, highlighting the power of the arguments. Our point is essentially what the reviewer has already concluded - random plus directed motility is a universal promoter of tumor development for reasons that can be traced to simple cell kinetics. One does not need to invoke higher-order processes e.g. angiogenesis to see these results. That said, the actual values we assumed for the frequency of symmetric division of stem cells (1%), the migration rate of tumor cells (15 cell widths per day), and the generational lifetimes of non-stem cells (*ρ *= 10) are reasonably in line with values determined elsewhere.

That tumor cells follow gradients has been known for some time. The notion has its origin as far back as Paget, who 120 years ago put forward the concept of "seed" and "soil" - that cancer cells metastasize nonrandomly to sites of growth. Since then it has been suspected that tumor cells "home" to specific sites. We now know that "chemokine receptors displayed on tumor cells allow the tumor cell to follow a gradient of chemokine to its target organ milieu that expresses the ligand for this chemokine receptor" [18]. It has even been shown that glioma cells follow chemorepellent gradients produced by the cells themselves, arguably to facilitate the freeing of space for better proliferation [19].

We agree that most progeny of cancer stem cells are non-stem cells. We stated that stem cells undergo "symmetric division" (i.e. reproduce themselves) at frequency p_s_. The rest of the time, their divisions would be asymmetric, i.e., produce a stem and a non-stem cell. We clarified this further in the manuscript by emphasizing that p_s _is low, and defining what symmetric and asymmetric division mean. We also define *ρ *as the generational lifetime of non-stem cells, and clarify that the estimate *ρ *= 10 is used. Thank you.

Our model, originally conceived, is quite different. The chemotactic stimuli in our model are not produced by the cells themselves but pre-exist in the milieu (in the spirit of Richmond et al.'s experimental observations [18]). Additionally, the basic premise of the Keller-Segel model is that the cell responds to fluctuations in estimates of the concentration of the critical substrate, rather than to the average concentration. By contrast, we assume the cell reads the local concentration gradient deterministically, but responds stochastically.

There are *S*+1 possible sites into which a cell may move in directed fashion (including the 'null movement'). Of the *S *nontrivial movements, we weight each by the factor *cos(angle θ between the gradient **and the movement direction **for each possible movement)*, for the sake of calculating the angle-weighted relative opportunity for movement in each available direction. The *cos(θ) *are summed over the *S *available sites to get *AS *(*A *is the average cosine value). Treating the effective "*cos(θ)*" of non-movement to be 1, the total sum of cosines is *AS*+1, where *AS *corresponds to actual movement. The total angle-weighted relative opportunity for movement (to one of the *S *sites) due to directed migration is thus *AS*/(*AS*+1) (we correct a misprint) where *A *is the average of the *cos(θ) *over these sites. This value, times the gradient strength ||, is now mapped to a probability of mobilization *G *through an arbitrary chemotactic/haptotactic responsiveness function H(z) for the cells, defined such that *H*(0) = 0 and *H *→ 1 monotonically as *z *→ ∞. The result is *G *= *H*{ ||*AS*/(*AS*+1)}. *G *is the probability the gradient succeeds in mobilizing the cell, and takes into account the proportion of movement possible and the strength of the gradient. Once mobilized, where the cell then goes is weighted by the respective cosines for movement, so that *cos(θ)G/(AS) *is the probability of movement to the site at angle *θ*. The probabilities of actual movement sum to *G*, and *1-G *is the probability there is no directed movement.

These points have been added to the paper to clarify the origin of *G*. Thank you.

### Reviewer 2: Glenn Webb, Department of Mathematics, Vanderbilt University, Nashville, Tennessee, USA

This paper makes a very strong case for the importance of differential cancer stem cell proliferation vs. committed cell proliferation in tumor invasive growth, independent of directed migration effects and independent of spatial heterogeneity. The results are established using agent based cellular automata models. Can similar results be obtained with continuum partial differential equations models?

### Authors' Response

We certainly ascribe to this general view, as we have before elaborated in a recent article [9]. We extend the concept here by showing that differential cancer stem cell proliferation vs. committed cell proliferation is merely advanced further by directed migration (except in the singular case where the point of attraction is in the tumor itself) in the same general manner as was seen with undirected migration without imposition of spatial heterogeneity.

In a follow-up collaboration we have indeed recapitulated these key results using continuum partial differential equations and hope to submit this work shortly.
